# Clusters of COVID-19 protective and risky behaviors and their associations with pandemic, socio-demographic, and mental health factors in the United States

**DOI:** 10.1016/j.pmedr.2021.101671

**Published:** 2021-12-14

**Authors:** Kristen Nishimi, Brian Borsari, Brian P. Marx, Raymond C. Rosen, Beth E. Cohen, Eleanor Woodward, David Maven, Paige Tripp, Ahmad Jiha, Joshua D. Woolley, Thomas C. Neylan, Aoife O'Donovan

**Affiliations:** a Mental Health Service, San Francisco Veterans Affairs Healthcare System, San Francisco, CA, USA; b Department of Psychiatry and Weill Institute for Neurosciences, University of California San Francisco, San Francisco, CA, USA; c National Center for PTSD, VA Boston Healthcare System, Boston, MA, USA; d Department of Psychiatry, Boston University School of Medicine, Boston, MA, USA; e Department of Medicine, University of California San Francisco, San Francisco, CA, USA; f Medical Service, San Francisco Veterans Affairs Healthcare System, San Francisco, CA, USA

**Keywords:** COVID-19, coronavirus disease 2019, PTSD, posttraumatic stress disorder, DASS, Depression Anxiety Stress Scale, PTSD Checklist-5, PCL-5, LCA, latent class analysis, BF, Bayes Factors, BIC, Bayesian Information Criterion, cAIC, consistent Akaike's Information Criterion, AWE, Approximate Weight of Evidence Criterion, mcaP, modal class assignment proportion, AvePP, average posterior class probability, OCC, odds of correct classification, ANOVA, analysis of variance, Risky behaviors, Protective behaviors, COVID-19, Mental health, Latent class analysis

## Abstract

Individual behaviors are critical for preventing the spread of coronavirus disease 2019 (COVID-19) infection. Given that both protective *and* risky behaviors influence risk of infection, it is critical that we understand how such behaviors cluster together and in whom. Using a data-driven approach, we identified clusters of COVID-19-related protective and risky behaviors and examined associations with socio-demographic, pandemic, and mental health factors. Data came from a cross-sectional online U.S. nationwide study of 832 adults with high levels of pre-pandemic trauma. Latent class analysis was performed with ten protective (e.g., washing hands, wearing masks) and eight risky (e.g., attending indoor restaurants, taking a flight) behaviors for COVID-19. Then, we examined distributions of socio-demographic and pandemic factors across behavior classes using ANOVA or Chi-square tests, and associations between mental health factors (depressive, anxiety, posttraumatic stress symptoms) and behavior classes using multinomial logistic regression. We identified four classes, including three classes with relatively low risky but high (28.8%), moderate (33.5%) and minimal (25.5%) protective behaviors and one high risky behaviors class with associated moderate protective behaviors (12.1%). Age, sexual orientation, political preference, and most pandemic factors differed significantly across behavior classes. Anxiety and posttraumatic stress symptoms, but not depression, were higher in the High Risk, but also Highly and Moderately Protective classes, relative to Minimally Protective. Prevention and intervention efforts should examine constellations of protective and risky behaviors to comprehensively understand risk, and consider current anxiety and posttraumatic stress symptoms as potential risk indicators.

## Highlights

• Protective and risky behaviors for COVID-19 cluster in a U.S.-based sample.
• Behavior classes had differing patterns of socio-demographics and pandemic exposure.
• Posttraumatic stress and anxiety were elevated among protective and risky classes.

## 1 Introduction

Health behaviors are critical for maintaining health and preventing infectious and other diseases. To maintain health, individuals must engage in protective behaviors (i.e., behaviors beneficial for health such as physical activity and medication adherence) and avoid risky behaviors (i.e., behaviors harmful for health such as smoking and excessive alcohol consumption). During the coronavirus disease 2019 (COVID-19) (Centers for Disease Control and Prevention, 2020) pandemic, individuals must pursue multiple protective behaviors (e.g., mask wearing) and avoid risky behaviors (e.g., socializing indoors). However, adherence to behavioral recommendations for preventing COVID-19 is variable (Hutchins, 2020), and we know little about how protective and risky behaviors cluster together – both within domains (e.g., engagement in all protective behaviors similarly) and between domains (e.g., engagement in protective while avoiding risky behaviors). Understanding how protective and risky behaviors cluster will inform tailored behavior change messaging and interventions.

Currently, we do not know how consistently people engage in protective and risky health behaviors. Prior studies of health-related behaviors typically examine a few individual behaviors in isolation (Liu et al., 2017), or indices of protective *or* risky behaviors separately (Rodriquez et al., 2018). Some evidence suggests risky health behaviors cluster together (Meader et al., 2016), but other studies indicate inconsistencies across patterns of protective and risky behaviors (Liang et al., 1999). Regarding infectious disease, most studies have focused on protective behaviors only (Weston et al., 2020, Bish and Michie, 2010). Absolute risk for adverse health outcomes relies on both protective and risky behaviors, and non-monotonic behavior patterns indicate there may be groups demonstrating different clusters of behavior (Weston et al., 2018). A better understanding of constellations of protective *and* risky behaviors can aid in identifying groups that may benefit most from specific interventions and guiding which messaging should be undertaken.

Mental health is a key factor influencing health behaviors (McLeroy et al., 1988). Psychiatric symptoms, including those associated with depression, anxiety, and posttraumatic stress disorder (PTSD), are associated with increases in risky behaviors like smoking (Liu et al., 2017) and decreases in preventive behaviors like healthcare utilization (Horenstein and Heimberg, 2020). Mental health is especially salient during the COVID-19 pandemic, which has been characterized by worsening mental health in general (Czeisler et al., 2020), and particularly among groups at elevated risk due to lifetime trauma or pre-existing psychiatric conditions (Vindegaard and Benros, 2020). Indeed, evidence indicates anxiety may increase preventive actions during infectious pandemics (Coughlin, 2012). Despite this, we know little about associations between mental health and COVID-19-related behaviors.

Data-driven clustering identifies groups with distinct behavior patterns. Only three studies to our knowledge have used clustering methods to examine COVID-19 protective behaviors (Kamenidou et al., 2020, Tomczyk et al., 2020, Wise et al., 2020), finding protective behavior clusters ranging from highly protective to risky. For example, one cluster analysis identified five population segments ranging from minimally to highly protective (Kamenidou et al., 2020), while another cluster analysis found three clusters with varying levels of compliance (Tomczyk et al., 2020). Women, older individuals, and those with higher socio-economic status tended to be overrepresented in the protective clusters (Kamenidou et al., 2020, Tomczyk et al., 2020), and higher perceived likelihood and severity of COVID-19 were associated with more protective clusters (Wise et al., 2020). Notably, no prior studies explicitly included risky COVID-19 behaviors, missing important compensatory or complementary patterns. Moreover, no studies have examined associations between psychiatric symptoms and COVID-19 behavior patterns. We estimated latent classes of COVID-19 protective and risky behaviors, and examined their associations with socio-demographics, pandemic factors, and mental health (i.e., depression, anxiety, posttraumatic stress) among a trauma-enriched community-based sample of U.S. adults. We hypothesized that we would: 1) identify multiple classes of COVID-19-related behaviors reflecting different behavioral patterns; 2) find associations between classes and socio-demographic and pandemic factors; and 3) find that classes demonstrating both higher risky and protective behavior engagement would show elevated psychiatric symptoms. Regarding socio-demographic and pandemic factors, based on prior evidence, we hypothesized that older age, female gender, and higher socio-economic status would be associated with lower risky and greater protective behavior.

## 2 Methods

The sample included community-dwelling U.S. adults who indicated interest in a treatment study for PTSD symptoms in 2017–2018 (Niles et al., 2020) and participated in a 30-minute online Qualtrics survey on COVID-19 experiences between August 4-September 19, 2020. Eligible individuals (age ≥ 18) provided electronic consent and received a $5 Amazon e-gift card upon survey completion. Of the 3,631 individuals who indicated interest in the 2017–2018 research study, 1,000 responded to the COVID-19 survey email invitation, and 896 consented and provided at least demographic information. The analytic sample included 832 individuals who had complete data on all variables. This study was approved and conducted in compliance with the Institutional Review Board at the University of California, San Francisco.

### 2.1 Measures

#### 2.1.1 COVID-19 protective and risky behaviors

Individuals reported the frequency of engagement in 18 behaviors in the past 30 days: 0 = never, 1 = rarely, 2 = sometimes, 3 = often, 4 = always. Ten behaviors were protective (i.e., washing hands, using hand sanitizer, wearing masks in public, maintaining six-foot distance from others, isolating from people outside one’s household, staying updated on COVID-19 news, sanitizing packages, stocking food or supplies, changing clothes after being outside one’s home, taking supplements for immunity) and eight were risky (i.e., going to the grocery store, using public transportation, taking a flight for vacation, gathering with others outdoors, gathering with others indoors, attending event with a large crowd, going to outdoor restaurants or bars, going to indoor restaurants or bars) with respect to COVID-19 infection.

#### 2.1.2 Socio-demographic factors

*Demographic* factors included age, gender, sexual orientation, and race/ethnicity. *Socio-economic* factors included education, employment, and income. *Political preference* was reported as Democrat, Republican, Independent, or Something else. *Family and residential* variables included marital status, living situation, residential area type, and U.S. region. See Table 3 for socio-demographic information.

#### 2.1.3 Pandemic factors

Individuals reported experiences since the pandemic began: having a COVID-19 test; having COVID-19; and having vulnerability factors for COVID-19, including health conditions (i.e., asthma; hypertension; kidney, lung, or liver disease; diabetes; blood or immune disorder; serious heart condition) and overweight/obese status. Pandemic factors also included COVID-19 in a household member, knowing anyone with COVID-19, providing COVID-19 care in employment, and past month frequency of going to one’s workplace. Individuals reported their likelihood of contracting COVID-19 in the next year (very unlikely, unlikely, neutral, likely, very likely) and severity of their symptoms if contracted (absent, mild, moderate, severe, extreme).

#### 2.1.4 Mental health

Past 30-day depressive and anxiety symptoms were assessed with the 21-item Depression Anxiety Stress Scale (DASS-21) (Antony et al., 1998), a validated, abbreviated version of DASS-42 (Lovibond and Lovibond, 1995). Seven-item subscales for depressive and anxiety symptoms were separately summed and multiplied by two to reflect DASS-42 subscale scores (Cronbach’s α_depression_ = 0.93, Cronbach’s α_anxiety_ = 0.89). Scores of ≥ 14 for depression and ≥ 10 for anxiety were considered “moderate” distress or higher, indicating potential clinically significant symptoms (Lovibond and Lovibond, 1995). Past 30-day PTSD severity in relation to one’s worst trauma was assessed with the 20-item PTSD Checklist*-5* (PCL-5), (Weathers et al., 2013) a widely used, validated self-report measure (Bovin et al., 2016). Total symptom severity sum scores were derived (Cronbach’s α = 0.96), as well as separate symptom cluster sum scores for intrusions, avoidance of trauma-related stimuli, negative alterations in cognition and mood, and alterations in arousal and reactivity. Total PCL-5 scores ≥ 33 indicated probable PTSD (Bovin et al., 2016).

### 2.2 Analyses

We used latent class analysis (LCA) to classify individuals into subgroups that capture heterogeneity in behavior engagement (Masyn, 2013, Nylund-Gibson and Choi, 2018). We used the *poLCA* package for R (Linzer and Lewis, 2011) to fit latent class models with 18 categorical behavior indicators (i.e., behavioral frequency variables), fitting models with one through eight classes. Models were estimated 50 times using maximum likelihood estimation with random initial parameters, selecting the lowest log-likelihood iteration. We examined fit criterion to determine the best fitting model. Relative goodness of fit was estimated with approximate Bayes Factor (BF), which compares the probabilities of k-class versus k + 1-class models being correct. Approximate BF > 10 is strong evidence for the k-class model (Masyn, 2013); data indicated evidence supporting the four-class and each higher-class model (Table 1). We examined commonly-assessed information criterion (i.e., Bayesian Information Criterion [BIC], consistent Akaike's Information Criterion [cAIC], Approximate Weight of Evidence Criterion [AWE]); lower values indicate better fit (Masyn, 2013, Nylund et al., 2007). Information criterion may not uniformly identify a single model, such as in our data (Table 1); BIC indicated a four-class, cAIC a three-class, and AWE a two-class model. We closely considered models with two to five classes, determining whether classes showed logical patterns, were distinct, could be readily labeled, and that no individual class had very few observations (Nylund-Gibson and Choi, 2018). Two and three class models had heterogeneous groups and approximate BF indicated poorer relative fit versus models with four or more classes. The five-class model included one very small class (proportion = 0.04). Moreover, studies suggest that BIC is superior to other information criterion (Nylund et al., 2007). Given all criteria, we determined the four-class model was most appropriate for the data.

**Table 1:** Model fit statistics for estimated latent class models for COVID-19 protective and risky behaviors.

N Classes	Log-likelihood	Approx. BF_k,k+1_	BIC	cAIC	AWE
1	−18030.95	<0.10	36546.02	36618.02	37246.13
2	−17194.38	<0.10	35363.72	35508.72	36773.68
3	−16695.06	<0.10	34855.91	35073.91	36975.70
4	−16435.16	>10	34826.96	35117.96	37656.59
5	−16200.59	>10	34848.65	35212.65	38388.13
6	−16009.01	>10	34956.33	35393.33	39205.65
7	−15890.35	>10	35209.85	35719.85	40169.00
8	−15749.19	–	35418.37	36001.37	41087.36

Approx. BF = Approximate Bayes Factor, BIC = Bayesian information criterion, cAIC = consistent Akaike’s Information Criterion, AWE = Approximate weight of evidence criterion

We inspected the four-class model classification diagnostics (Table 2): relative entropy (overall posterior classification precision), modal class assignment proportions (mcaP_k_; proportion of individuals modally assigned to class k), average posterior class probabilities (AvePP_k_; average class k posterior class probabilities among individuals with modal class k), and odds of correct classification (OCC_k_; odds of correct classification to k based on modal class assignment, >5 = high accuracy) (Masyn, 2013). We defined modal class assignment, classifying each individual into one of four classes based on their highest posterior probability. There was good overall class separation (relative entropy = 0.87), high probabilities for observations assigned to the modal class (AvePP_k_s 0.90–0.96), and good assignment accuracy (OCC_k_s > 5) (Masyn, 2013). We therefore determined modal class assignment was a feasible indicator.

**Table 2:** Model classification statistics for the four-class model of COVID-19 protective and risky behaviors (relative entropy = 0.87).

Class k	π	SE_π_	mcaP	AvePP	OCC
1	0.29	0.01	0.29	0.94	38.23
2	0.33	0.02	0.34	0.90	18.26
3	0.12	0.02	0.12	0.96	172.72
4	0.26	0.02	0.25	0.94	45.27

π = estimated proportion, SE = standard error, mcaP = modal class assignment proportion, AvePP = average posterior class probability, OCC = odds of correct classification.

**Table 3:** Distribution of socio-demographic and pandemic factors by COVID-19 protective and risky behavior latent classes (n = 832).

		Full Sample	Highly Protective	Moderately Protective	Minimally Protective	Risky	ANOVA or Chi-Square
		n = 832	n = 240, 28.8%	n = 279, 33.5%	n = 212, 25.5%	n = 101, 12.1%	
Correlate		*N (%)*	*N (%)*	*N (%)*	*N (%)*	*N (%)*	*p-value*
Socio-Demographics							0.037
Age (*m (SD)*), in years		37.0 (11.0)	38.2 (11.0)	37.5 (11.7)	35.8 (10.7)	35.2 (9.0)	
Gender	Man	167 (20.1)	44 (26.3)	48 (28.7)	49 (29.3)	26 (15.6)	0.18
	Woman	644 (77.4)	191 (29.7)	221 (34.3)	157 (24.4)	75 (11.6)	
	Non-Binary/Trans gender/Other	21 (2.5)	5 (23.8)	10 (47.6)	6 (28.6)	0 (0.0)	
Sexual Orientation	Heterosexual	660 (79.3)	189 (28.6)	216 (32.7)	163 (24.7)	92 (13.9)	0.040
	Homosexual	53 (6.4)	16 (30.2)	15 (28.3)	19 (35.8)	3 (5.7)	
	Bisexual/Queer/Pansexual/Other	119 (14.3)	35 (29.4)	48 (40.3)	30 (25.2)	6 (5.0)	
Race/Ethnicity	Non-Hispanic White	488 (58.7)	122 (25.0)	166 (34.0)	138 (28.3)	62 (12.7)	0.14
	Black	112 (13.5)	43 (38.4)	33 (29.5)	19 (17.0)	17 (15.2)	
	Asian	76 (9.1)	27 (35.5)	23 (30.3)	18 (23.7)	8 (10.5)	
	Latinx	75 (9.0)	26 (34.7)	27 (36.0)	16 (21.3)	6 (8.0)	
	Other^a^/More Than One Race	81 (9.7)	22 (27.2)	30 (37.0)	21 (25.9)	8 (10.0)	
Educational Attainment	High School or Less	77 (9.3)	24 (31.2)	25 (32.5)	19 (24.7)	9 (11.7)	0.42
	Some College/2-yr College Degree	230 (27.6)	79 (34.3)	75 (32.6)	51 (22.2)	25 (10.9)	
	4-yr College Degree/Grad School	525 (63.1)	137 (26.1)	179 (34.1)	142 (27.0)	67 (12.8)	
Current Employment Status	Employed Full Time	466 (56.0)	132 (28.3)	163 (35.0)	106 (22.7)	65 (13.9)	0.43
	Employed Part Time	127 (15.3)	31 (24.4)	44 (34.6)	39 (30.7)	13 (10.2)	
	Unemployed	171 (20.6)	51 (29.8)	54 (31.6)	50 (29.2)	16 (9.4)	
	Student	33 (4.0)	12 (36.4)	10 (30.3)	9 (27.3)	2 (6.1)	
	Retired	19 (2.3)	6 (31.6)	6 (31.6)	5 (26.3)	2 (10.5)	
	Furloughed	16 (1.9)	8 (50.0)	2 (12.5)	3 (18.8)	3 (18.8)	
Annual Household Income	≤$50,000/year	344 (41.3)	98 (28.5)	109 (31.7)	104 (30.2)	33 (9.6)	0.051
	$50,001-$100,000/year	329 (39.5)	100 (30.4)	104 (31.6)	77 (23.4)	48 (14.6)	
	$100,001-$150,000/year	102 (12.3)	27 (26.5)	42 (41.2)	17 (16.7)	16 (15.7)	
	>$150,000/year	57 (6.9)	15 (26.3)	24 (42.1)	14 (24.6)	4 (7.0)	
Political Preference	Republican	123 (14.8)	28 (22.8)	30 (24.4)	27 (22.0)	38 (30.9)	<0.001
	Democrat	434 (52.2)	131 (30.2)	157 (36.2)	107 (24.7)	39 (9.0)	
	Independent	186 (22.4)	57 (30.6)	62 (33.3)	49 (26.3)	18 (9.7)	
	Something Else	88 (10.6)	24 (27.3)	30 (34.1)	29 (33.0)	5 (5.7)	
Marital status	Married	280 (33.7)	92 (32.9)	93 (33.2)	58 (20.7)	37 (13.2)	0.39
	Single, In a Relationship	250 (30.0)	69 (27.6)	82 (32.8)	65 (26.0)	34 (13.6)	
	Single, No Relationship	237 (28.5)	61 (25.7)	83 (35.0)	71 (30.0)	22 (9.3)	
	Separated/Divorced/Widowed	65 (7.8)	18 (27.7)	21 (32.3)	18 (27.7)	8 (12.3)	
Living Situation	Living with Others (versus Alone)	651 (78.2)	194 (29.8)	224 (34.4)	161 (24.7)	72 (11.1)	0.16
Live with Children	Living with Children	284 (34.1)	93 (32.7)	88 (31.0)	58 (20.4)	45 (15.8)	0.006
Housing Type	House/Condominium	535 (64.3)	158 (29.5)	184 (34.4)	127 (23.7)	66 (12.3)	0.73
	Apartment	285 (34.3)	79 (27.7)	92 (32.6)	80 (28.1)	34 (11.9)	
	Other	12 (1.4)	3 (25.0)	3 (25.0)	5 (41.7)	1 (8.3)	
Residential Area Type	Urban	421 (50.6)	138 (33.3)	129 (30.6)	98 (23.3)	56 (13.3)	0.057
	Suburban	296 (35.6)	70 (23.6)	109 (36.8)	79 (26.6)	38 (12.8)	
	Town	65 (7.8)	16 (24.6)	24 (36.9)	23 (35.4)	2 (3.1)	
	Rural	50 (6.0)	16 (32.0)	17 (34.0)	12 (24.0)	5 (10.0)	
Region of Residence	West	272 (32.7)	70 (25.7)	98 (36.0)	74 (27.2)	30 (11.0)	0.25
	Midwest	138 (16.6)	48 (34.8)	37 (26.8)	38 (27.5)	15 (10.9)	
	Northeast	165 (19.8)	49 (29.7)	63 (38.2)	37 (22.4)	16 (9.7)	
	South	257 (30.9)	73 (28.4)	81 (31.5)	63 (24.5)	40 (15.6)	

Pandemic Factors							
Had a COVID-19 Test	Yes	268 (32.2)	73 (27.2)	103 (38.4)	59 (22.0)	33 (12.3)	0.17
Had COVID-19	Yes, Diagnosed with Test	11 (1.3)	5 (45.5)	3 (27.3)	0 (0.0)	3 (27.3)	0.034
	Probably, Diagnosed without Test	9 (1.1)	1 (11.1)	3 (33.3)	2 (22.2)	3 (33.3)	
	Maybe, Suspected COVID-19	135 (16.2)	29 (21.5)	57 (42.2)	31 (23.0)	18 (13.3)	
	No COVID-19	677 (81.4)	205 (30.3)	216 (31.9)	179 (26.4)	77 (11.4)	
Vulnerable Conditions	Yes	284 (34.1)	90 (31.7)	105 (37.0)	57 (20.1)	32 (11.3)	0.047
Overweight or Obese	Yes	372 (44.7)	114 (30.6)	128 (34.4)	88 (23.7)	42 (11.3)	0.53
COVID-19 in a Household Member	Yes, Diagnosed with Test	28 (3.4)	9 (32.1)	6 (21.4)	6 (21.4)	7 (25.0)	0.004
	Probably, Diagnosed without Test	6 (0.7)	2 (33.3)	1 (16.7)	0 (0.0)	3 (50.0)	
	Maybe, Suspected COVID-19	80 (9.6)	13 (16.3)	37 (46.3)	19 (23.8)	11 (13.8)	
	No Household COVID-19	718 (86.3)	216 (30.1)	235 (32.7)	187 (26.0)	80 (11.1)	
Know Anyone with COVID-19	Yes	503 (60.5)	159 (31.6)	187 (37.2)	110 (21.9)	47 (9.3)	<0.001
COVID-19 Care in Employment	Direct Care	33 (4.0)	7 (21.2)	15 (45.5)	4 (12.1)	7 (21.2)	
	Supportive Care	52 (6.2)	14 (26.9)	20 (38.5)	9 (17.3)	9 (17.3)	
	No COVID-19 Care	747 (89.8)	219 (29.3)	244 (32.7)	199 (26.6)	85 (11.4)	
Went into one’s Workplace in the past month	Never	374 (45.0)	138 (36.9)	104 (27.8)	106 (28.3)	26 (7.0)	<0.001
	Rarely	109 (13.1)	27 (24.8)	48 (44.0)	20 (18.3)	14 (12.8)	
	Sometimes	84 (10.1)	14 (16.7)	32 (38.1)	23 (27.4)	15 (17.9)	
	Often	98 (11.8)	24 (24.5)	33 (33.7)	23 (23.5)	18 (28.4)	
	Always	167 (20.1)	37 (22.2)	62 (37.1)	40 (24.0)	28 (16.8)	
Likelihood of COVID-19, *m (SD)*	Very Unlikely = 1 to Very Likely = 5	2.7 (1.0)	2.6 (1.1)	2.8 (0.9)	2.7 (0.9)	2.5 (1.2)	0.001
Severity of COVID-19, *m (SD)*	Absent = 1 to Extreme Symptoms = 5	2.6 (0.9)	2.9 (1.0)	2.6 (0.9)	2.5 (0.9)	2.3 (1.0)	<0.001
^a^ Other includes Native Hawaiian, Pacific Islander, American Indian, Alaska Native, and Middle Eastern							

We estimated bivariate associations between socio-demographics and pandemic factors with class assignment using Chi-square and ANOVA tests. We estimated multinomial logistic regression models with class assignment as the outcome. To examine linear associations and threshold effects, we separately modeled continuous symptoms and binary mental health indicators as independent variables. Models first adjusted for socio-demographics, then additionally for pandemic factors, as COVID-19 experiences and perceived likelihood of contracting COVID-19 may be associated with behaviors (de Bruin and Bennett, 2020).

## 3 Results

The sample was predominantly female (77.4%) and racially/ethnically diverse with a mean age 37 (SD = 11.0) (Table 3). There was a range of income, employment status, and family and residential circumstances, but 63.1% had a 4-year college degree or greater. Regarding political preference, 50.2% identified as Democrat, 14.8% as Republican, 22.4% as Independent, and 10.6% as something else. Individuals came from 48 states plus Washington D.C. in the West (32.7%), Midwest (16.6%), Northeast (19.8%), and South (30.9%) of the U.S.

Regarding COVID-19 experiences, 32.2% of the sample had a COVID-19 test, 2.4% were diagnosed with COVID-19 with a test, and 16.2% suspected they had COVID-19 (Table 3). There was variation in perceived likelihood of contracting COVID-19 (15.5% reported very unlikely, 3.0% very likely) and severity of symptoms if contracted (12.9% reported no symptoms, 2.8% extreme symptoms).

The sample had high levels of depressive (m = 13.6 [SD = 11.9]), anxiety (m = 9.9 [SD = 9.9]), and PTSD (m = 24.5 [SD = 19.9]) symptoms, consistent with recruitment of people experiencing trauma-related psychological symptoms in 2017/2018 (Niles et al., 2020). Using diagnostic cutoffs, 44.2% had elevated depression, 43.4% had elevated anxiety, and 32.6% had probable PTSD. Mental health scores were highly correlated (r_depression-anxiety_ = 0.71; r_depression-PTSD_ = 0.71; r_anxiety-PTSD_ = 0.70).

### 3.1 COVID-19 behavior classes

**Fig. 1:**
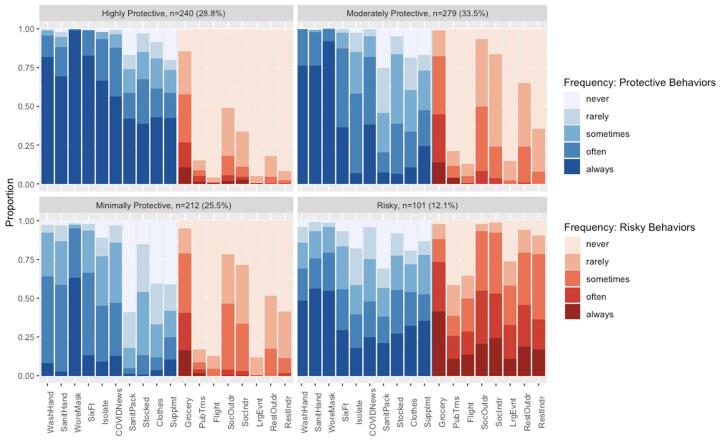
Distribution of behavior frequencies across COVID-19 protective and risky behavior latent classes. Proportions of past 30-day frequency of individual protective and risky behaviors across four COVID-19 behavior latent classes.

**Fig. 2:**
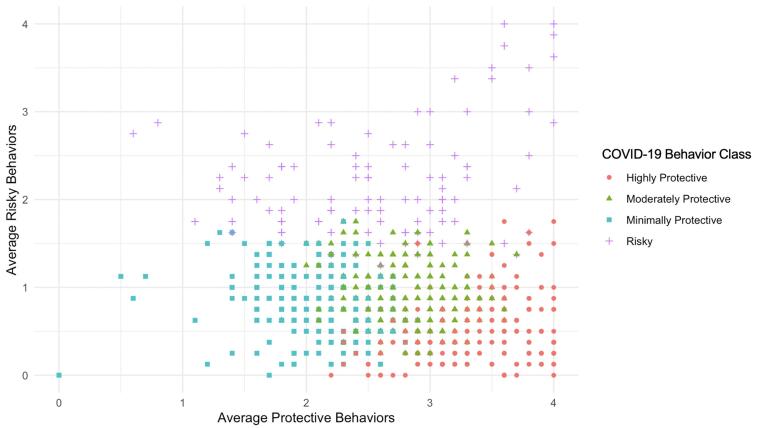
Distribution of COVID-19 protective and risky behavior latent classes by average protective and risky behavior engagement. Distribution of four COVID-19 behavior latent classes by average protective and average risky behaviors (derived as averaged frequency 0 = never to 4 = always across 10 protective behavior items and 8 risky behavior items, separately).

We examined the distribution of behaviors across classes (Fig. 1). The first three classes engaged in low levels of risky behavior, but differed in levels of protective behaviors (Fig. 2). One quarter of the sample (25.5%) fell into a group labeled “Minimally Protective” with low protective behavior endorsement, but also low-moderate risky behavior endorsement. This class had high endorsement of always wearing masks, but low levels of other protective behaviors. Although they tended to report never engaging in risky behaviors involving the public or crowds (e.g., taking a flight for vacation, attending a large event), they sometimes engaged in other risky behaviors (e.g., socializing and eating at restaurants). The second most protective and largest class (33.5%) was labeled “Moderately Protective”, characterized by high endorsement of always engaging in recommended sanitary protective behaviors (e.g., washing and sanitizing hands, wearing masks) and moderate endorsement of other protective behaviors. This class did not engage in public/crowd-related risky behaviors, but did endorse other socializing activities. The group characterized by the highest level of protective behaviors (28.8%) was labeled “Highly Protective”. This class had high endorsement of always engaging in all protective behaviors and never engaging in most risky behaviors. The smallest class (12.1%) was labeled “Risky”. This class had moderate endorsement of always washing and sanitizing hands and wearing masks, but inconsistent engagement in other protective behaviors. They reported the highest levels of all risky behaviors.

### 3.2 Socio-demographics, pandemic factors, and COVID-19 behavior classes

Age, sexual orientation, political preference, and living with children were differentially distributed across COVID-19 behavior classes (Table 3). Protective classes were similar socio-demographically, whereas Risky was most distinctive. The Risky class was younger, had the highest proportions of heterosexual and Republican individuals, and was most likely to live with children.

All pandemic factors were associated with behavior classes, apart from having a COVID-19 test, overweight/obese status, and providing care for individuals with COVID-19. The Minimally Protective class had low proportions of COVID-19 infection and moderate perceived likelihood and severity of COVID-19. The Moderately Protective class had high proportions of suspected COVID-19 and the highest perceived likelihood of contracting COVID-19. The Highly Protective class had the lowest proportion of COVID-19 infection and highest perceived severity of COVID-19. The Risky class was most likely to have had COVID-19 but reported the lowest perceived likelihood and severity of COVID-19.

### 3.3 Mental health and COVID-19 behavior classes

As the Minimally Protective class had low risky *and* protective behaviors, it served as the reference group for multinomial logistic regressions. Depression was not associated with differential assignment in behavior classes. Adjusting for socio-demographics, one standard deviation increase in anxiety symptoms was associated with 1.43 (95%CI 1.10–1.86) times higher odds of being in the Risky versus Minimally Protective class (Table 4). Clinically elevated anxiety was associated with higher odds of being in the Risky (OR = 2.63, 95%CI 1.52–4.54), Moderately (OR = 1.88, 95%CI 1.26–2.81) and Highly Protective (OR = 1.58, 95%CI 1.04–2.40) classes versus Minimally Protective. Associations were attenuated but largely remained significant when adjusting for pandemic factors.

**Table 4:** Multinomial logistic regression for mental health predicting COVID-19 protective and risky behavior classes (n = 832)

	Highly Protective, n = 240 (28.8%)		Moderately Protective, n = 279 (33.5%)		Risky, n = 101 (12.1%)	
Predictor	*OR (95%CI)*	*p-value*	*OR (95%CI)*	*p-value*	*OR (95%CI)*	*p-value*
*Adjusted for Socio-Demographics*						
Depressive Symptoms	1.03 (0.84–1.27)	0.77	1.02 (0.83–1.24)	0.88	1.15 (0.88–1.49)	0.30
Clinically Elevated Depression	1.01 (0.67–1.52)	0.97	1.10 (0.74–1.62)	0.64	1.50 (0.89–2.55)	0.13
Anxiety Symptoms	1.21 (0.98–1.50)	0.076	1.22 (0.99–1.50)	0.060	1.43 (1.10–1.86)	0.008
Clinically Elevated Anxiety	1.58 (1.04–2.40)	0.034	1.88 (1.26–2.81)	0.002	2.63 (1.52–4.54)	0.001
PTSD Symptoms	1.28 (1.04–1.58)	0.022	1.21 (0.99–1.49)	0.060	1.48 (1.13–1.92)	0.004
Intrusions	1.36 (1.10–1.69)	0.005	1.35 (1.09–1.66)	0.005	1.67 (1.28–2.19)	<0.001
Avoidance	1.27 (1.03–1.56)	0.023	1.11 (0.91–1.36)	0.29	1.17 (0.89–1.53)	0.25
Negative Cognitions and Mood	1.15 (0.93–1.41)	0.19	1.08 (0.88–1.31)	0.47	1.32 (1.01–1.72)	0.039
Arousal and Reactivity	1.29 (1.04–1.59)	0.019	1.27 (1.04–1.55)	0.022	1.49 (1.15–1.94)	0.003
Probable PTSD	2.18 (1.39–3.43)	0.001	1.81 (1.17–2.79)	0.008	3.46 (1.96–6.12)	<0.001

*Adjusted for Socio-Demographics and Pandemic Factors*						
Depressive Symptoms	0.91 (0.72–1.14)	0.42	0.95 (0.77–1.16)	0.60	1.09 (0.82–1.46)	0.54
Clinically Elevated Depression	0.81 (0.52–1.27)	0.36	1.00 (0.66–1.50)	0.99	1.52 (0.85–2.69)	0.16
Anxiety Symptoms	1.11 (0.88–1.41)	0.37	1.10 (0.89–1.38)	0.38	1.36 (1.02–1.83)	0.039
Clinically Elevated Anxiety	1.34 (0.85–2.12)	0.21	1.59 (1.04–2.43)	0.033	2.45 (1.35–4.44)	0.003
PTSD Symptoms	1.18 (0.94–1.49)	0.15	1.14 (0.92–1.41)	0.23	1.43 (1.07–1.91)	0.015
Intrusions	1.30 (1.03–1.63)	0.027	1.27 (1.02–1.58)	0.030	1.60 (1.20–2.12)	0.001
Avoidance	1.25 (1.00–1.56)	0.052	1.07 (0.87–1.32)	0.51	1.12 (0.83–1.50)	0.45
Negative Cognitions and Mood	1.05 (0.83–1.31)	0.70	1.01 (0.82–1.24)	0.95	1.27 (0.96–1.69)	0.097
Arousal and Reactivity	1.17 (0.93–1.48)	0.17	1.19 (0.96–1.48)	0.11	1.48 (1.11–1.96)	0.007
Probable PTSD	1.92 (1.18–3.12)	0.009	1.62 (1.03–2.54)	0.037	3.25 (1.77–6.00)	<0.001
Outcome reference: Minimally Protective, n = 212 (25.5%); Symptoms are standardized.						

Higher PTSD severity was associated with higher odds of being in the Risky (OR = 1.48, 95%CI 1.13–1.92) and Highly Protective (OR = 1.28, 95%CI 1.04–1.58) classes versus Minimally Protective. Probable PTSD was associated with higher odds of being in the Risky (OR = 3.46, 95%CI 1.96–6.12), Moderately (OR = 1.81, 95%CI 1.17–2.79) and Highly Protective (OR = 2.18, 95%CI 1.39–3.43) classes versus Minimally Protective. Adjusting for pandemic factors attenuated associations. Intrusion and arousal symptom clusters followed similar patterns as total PTSD symptom severity, while avoidance was associated with being in the Highly Protective and negative cognitions and mood with being in the Risky class.

## 4 Discussion

To our knowledge, we have conducted the first LCA to elucidate patterns of COVID-19 protective and risky behaviors, a critical first step towards understanding and promoting optimal behavioral strategies. We identified four behavior classes in our geographically dispersed sample, including Minimally, Moderately, and Highly Protective, with relatively low risky but varying levels of protective behaviors, and Risky, which comprised a minority of participants but reported high levels of risky behaviors combined with moderate protective behaviors. Although protective behaviors may compensate for risky behaviors, it is likely impossible to mitigate all risks associated with highly risky activities. Assessing risky in addition to protective behaviors was critical to identifying this group. We also identified socio-demographic, pandemic-related, and mental health correlates of class membership, which underscore the potential for population segmentation approaches to public health (Yan et al., 2018).

Prior studies examining protective COVID-19 behaviors identified groups ranging from protective/cautious to risky. Among 3,359 Greek adults, cluster analyses of 27 preventive behaviors identified five population segments ranging from “Meticulous Proactive” to “Unconcerned Citizens” (Kamenidou et al., 2020). Another study of 157 German young adults identified a three-class structure across nine recommended behaviors: low compliance, high compliance, and public compliance (e.g., social distancing, but low avoidance of facial touching) (Tomczyk et al., 2020). Finally, cluster analyses among 1,591 U.S. adults identified 16 subgroups ranging from low to high engagement across four behaviors: avoiding social interaction, hand washing, staying home, and traveling less (Wise et al., 2020). We extend prior studies by identifying a “risky” class, underscoring the importance of explicitly assessing both protective and risky behaviors to recognize compensatory patterns of behavior.

Several socio-demographic factors differed across COVID-19 behavior classes. The Risky class tended to be younger, consistent with prior studies (Kamenidou et al., 2020, Tomczyk et al., 2020). We did not find gender differences in behavior classes, like some (Wise et al., 2020), but not all prior work (Kamenidou et al., 2020, Tomczyk et al., 2020); our predominantly female sample may have precluded identifying such differences. Sexual orientation differed across behavior classes, with the Risky class including relatively more heterosexual individuals. In contrast, prior work found sexual minority individuals performed fewer COVID-19 protective behaviors than heterosexual individuals (Ko et al., 2020, Solomon et al., 2021). Sexual orientation was not associated with behavior classes in our adjusted models, therefore bivariate associations may have been confounded by other socio-demographic factors. COVID-19 behavior classes were largely not patterned by racial/ethnic identity or socio-economic status. This was surprising as higher socio-economic position has been associated with greater self-protective COVID-19 behaviors (Papageorge et al., 2021), potentially because those with lower resources experience circumstances where protective behaviors are more difficult, like essential work. Lack of racial/ethnic differences was more consistent with emerging evidence - racial/ethnic differences in COVID-19 preventive behaviors are mixed (Sauceda et al., 2020), in stark contrast to racial/ethnic differences in risk for *contracting* COVID-19 (Mackey et al., 2021), which requires further study. Political preference was strongly patterned by COVID-19 behavior class, with the Risky class most likely to identify as Republican. Other U.S. data indicate that Republican preference is associated with less protective COVID-19 behavior, potentially due to separate news sources, media polarization, or collectivist versus individualist views (Bruine de Bruin et al., 2020). Notably, our non-representative sample included a larger proportion of Democrats (50% versus 30%) and smaller proportion of Republicans (15% versus 29%) than the U.S. population (Gallup Inc., 2020).

COVID-19 experiences and perceptions were related to COVID-19 behavior classes. Higher protective classes had some COVID-19 infection, higher vulnerability, and higher perceived likelihood and severity of infection. The Risky class reported the highest proportion of COVID-19 infections, but also lowest perceived likelihood and severity of infection. Given cross-sectional data, it is unclear whether COVID-19-related exposures or perceptions influenced behaviors or vice versa. However, patterns suggest that neither objective exposures to, nor subjective perceptions of, COVID-19 alone are sufficient to increase protective and reduce risky behaviors. Instead, COVID-19 exposures and perceptions likely interact with other contextual and individual factors.

Anxiety and PTSD, but not depression, were associated with greater odds of being in the Risky as well as Moderately and Highly Protective classes compared to Minimally Protective. The Minimally Protective class, which had the lowest anxiety and PTSD symptoms, potentially reflected a low-risk context where frequent protective behaviors were unnecessary. Elevated anxiety and PTSD symptoms may influence greater risky behavior in some individuals (i.e., Risky) and greater protective behaviors in others (i.e., Moderately and Highly Protective). Anxiety and PTSD are associated with emotion dysregulation and impulsivity, (Zimmermann, 2010) contributing to riskier behaviors. Indeed, risky or destructive behavior is a PTSD symptom (Weathers et al., 2013). Conversely, worry, threat sensitivity, and hypervigilance in anxiety and PTSD could increase preventive behaviors (Venkateswaran and Hauser, 2020). For example, state anxiety was associated with more preventive measures during previous epidemics (Weston et al., 2018). Although depression has been linked with riskier health behaviors (Liu et al., 2017, Rodriquez et al., 2018), few studies have examined depression and infectious disease-related behaviors and it was unassociated in our study. There may be trade-offs whereby those at lower COVID-19 infection risk due to protective behavioral patterns (e.g., Highly Protective), may also experience poorer mental health. While prospective research is needed, behavioral health practitioners should be aware of potential associations between poorer mental health with highly risky *and* protective behavior.

Behavior change efforts will be most effective when appropriately targeted. As first steps, researchers must demonstrate distinct behavioral classes within the population, and that classes differ by key characteristics. Our findings may inform data-driven segmentation of populations to: 1) develop relevant behavior change messaging (e.g., behavior maintenance for protective groups, increases in protective *and* decreases in risky behaviors for risky groups); and 2) recognize group characteristics to identify individuals and target messaging modalities (Yan et al., 2018). Effective tailored messaging addresses individual needs and personal relevance, and data-driven clustering can guide how to focus limited resources to optimize outcomes. Messages should address the full spectrum of behaviors (i.e., risky and protective, versus only protective), and targeted messages may recommend decreasing risk while acknowledging current engagement in protective behaviors.

Our study has several limitations. Data were cross-sectional; therefore, we cannot draw causal conclusions. We hypothesize that mental health influences COVID-19 behavior; it is possible behaviors could influence mental health as there are reciprocal relations among affect, cognitions, and behaviors. Self-reported measures were subject to reporting biases, particularly recommended prevention behaviors. We cannot generalize beyond the U.S.-based sample enriched for trauma and probable PTSD. Additionally, our behavior classes reflect this sample and may not generalize. Despite this, our classes are relevant for understanding potential risky and protective behavior constellations that may occur in the broader population.

Developing effective behavioral interventions requires consideration of behavioral patterns more holistically, including both protective and risky behaviors. Socio-demographic differences in behavior patterns may reflect individual determinants and contextual factors that enable protective behaviors or necessitate risky behaviors. Our data indicate elevated anxiety and PTSD symptoms might be relevant indicators for engagement in both heightened risk and protective behaviors amid the pandemic. To maximize the behavioral defense against COVID-19 and other infectious diseases, we must pay attention to the non-monotonic nature of health behaviors.

## Funding

This work was supported by the UCSF Department of Psychiatry Rapid Award (AOD) and the National Institutes of Mental Health (AOD; K01MH109871). KN was supported by the Department of Veterans Affairs Office of Academic Affiliations Advanced Fellowship Program in Mental Illness Research and Treatment, the Medical Research Service of the SFVAHCS, and the Department of Veterans Affairs Sierra-Pacific Mental Illness Research, Education, and Clinical Center (MIRECC).

### CRediT authorship contribution statement

**Kristen Nishimi:** Conceptualization, Methodology, Data curation, Formal analysis, Software, Visualization, Investigation, Writing – original draft. **Brian Borsari:** Conceptualization, Methodology, Funding acquisition, Writing – review & editing. **Brian P. Marx:** Conceptualization, Methodology, Funding acquisition, Writing – review & editing. **Raymond C. Rosen:** Conceptualization, Methodology, Funding acquisition, Writing – review & editing. **Beth E. Cohen:** Conceptualization, Methodology, Funding acquisition, Writing – review & editing. **Eleanor Woodward:** Conceptualization, Methodology, Writing – review & editing. **David Maven:** Conceptualization, Methodology, Investigation, Writing – review & editing. **Paige Tripp:** Conceptualization, Methodology, Investigation, Writing – review & editing. **Ahmad Jiha:** Conceptualization, Methodology, Investigation, Writing – review & editing. **Joshua D. Woolley:** Conceptualization, Methodology, Writing – review & editing. **Thomas C. Neylan:** Conceptualization, Methodology, Writing – review & editing. **Aoife O'Donovan:** Conceptualization, Methodology, Funding acquisition, Investigation, Project administration, Resources, Supervision, Validation, Writing – original draft.

## Declaration of Competing Interest

The authors declare that they have no known competing financial interests or personal relationships that could have appeared to influence the work reported in this paper.
